# Patient-reported advantages and disadvantages of peritoneal dialysis: results from the PDOPPS

**DOI:** 10.1186/s12882-019-1304-3

**Published:** 2019-04-02

**Authors:** Nidhi Sukul, Junhui Zhao, Douglas S. Fuller, Angelo Karaboyas, Brian Bieber, James A. Sloand, Lalita Subramanian, David W. Johnson, Matthew J. Oliver, Kriang Tungsanga, Tadashi Tomo, Rachael L. Morton, Hal Morgenstern, Bruce M. Robinson, Jeffrey Perl, on behalf of the clinical application of PD therapy working group

**Affiliations:** 1 Michigan Medicine, 1500 E. Medical Center Dr., SPC 5364, Ann Arbor, Michigan 48109-5364 USA; 2 Arbor Research Collaborative for Health, Ann Arbor, MI USA; 3 Baxter Healthcare Corporation, Deerfield, IL USA; 4 Centre for Kidney Disease Research, University of Queensland at Princess Alexandra Hospital, Brisbane, QLD Australia; 5 Sunnybrook Health Sciences Centre, Toronto, ON Canada; 6 King Chulalongkorn Memorial Hospital, Bangkok, Thailand; 7 Oita University Hospital, Yufu, Japan; 8 NHMRC Clinical Trials Centre, The University of Sydney, Sydney, NSW Australia; 9 Departments of Epidemiology and Environmental Health Sciences, School of Public Health, and Department of Urology, Medical School, University of Michigan, Ann Arbor, MI USA; 10 St. Michael’s Hospital, Toronto, ON Canada

**Keywords:** Depression, Patient-reported measures, Patient selection, Peritoneal dialysis, Quality of life, Surveys and questionnaires, Technique survival

## Abstract

**Background:**

Patient-reported measures are increasingly recognized as important predictors of clinical outcomes in peritoneal dialysis (PD). We sought to understand associations between patient-reported perceptions of the advantages and disadvantages of PD and clinical outcomes.

**Methods:**

In this cohort study, 2760 PD patients in the Peritoneal Dialysis Outcomes and Practice Patterns Study (PDOPPS) completed a questionnaire on their PD experience, between 2014 and 2017. In this questionnaire, PDOPPS patients rated 17 aspects of their PD experience on a 5-category ordinal scale, with responses scored from − 2 (major disadvantage) to + 2 (major advantage). An advantage/disadvantage score (ADS) was computed for each patient by averaging their response scores. The ADS, along with each of these 17 aspects, were used as exposures. Outcomes included mortality, transition to hemodialysis (HD), patient-reported quality of life (QOL), and depression. Cox regression was used to estimate associations between ADS and mortality, transition to HD, and a composite of the two. Logistic regression with generalized estimating equations was used to estimate cross-sectional associations of ADS with QOL and depression.

**Results:**

While 7% of PD patients had an ADS < 0 (negative perception of PD), 59% had an ADS between 0 and < 1 (positive perception), and 34% had an ADS ≥1 (very positive perception). Minimal association was observed between mortality and the ADS. Compared with a very positive perception, patients with a negative perception had a higher transition rate to HD (hazard ratio [HR] = 1.67; 95% confidence interval [CI]: 1.21, 2.30). Among individual items, “space taken up by PD supplies” was commonly rated as a disadvantage and had the strongest association with transition to HD (HR = 1.28; 95% CI 1.07, 1.53). Lower ADS was strongly associated with worse QOL rating and greater depressive symptoms.

**Conclusions:**

Although patients reported a generally favorable perception of PD, patient-reported disadvantages were associated with transition to HD, lower QOL, and depression. Strategies addressing these disadvantages, in particular reducing solution storage space, may improve patient outcomes and the experience of PD.

**Electronic supplementary material:**

The online version of this article (10.1186/s12882-019-1304-3) contains supplementary material, which is available to authorized users.

## Background

In the United States (US), changes in dialysis reimbursement policies have led to unprecedented growth in the use of peritoneal dialysis (PD) since 2011 [1]. Compared with facility hemodialysis (HD), PD is more cost-effective [2, 3], is less technically demanding [4], minimizes the exposure of patients to hospital-acquired infections [5], is more feasible in rural and remote settings [6], and is associated with better preservation of residual kidney function [7, 8] – a factor associated with survival advantage among patients receiving dialysis [9–12]. Commonly perceived patient advantages of PD include enhanced opportunities for rehabilitation and return to employment and improved satisfaction and quality of life (QOL) [13]. Studies have suggested that reasons patients select PD include less interference with lifestyle, preference to be independent, wanting to dialyze at night, and less requirement for travel for dialysis treatments [13, 14]. However, patients also view disadvantages to PD therapy, including “catheter care,” “high frequency of dialysis in a day,” and “troubling other people” [15]. Negative aspects of PD have been cited as “problem with supplies,” “frequency/length of treatment,” “bloating/pain,” “interference with sleep,” and “change in daily routine” [16].

To help patients make an informed decision about whether to pursue PD, nephrologists and other health educators typically explain presumptive advantages and disadvantages of this dialysis modality. However, there is little insight into how patients performing PD typically rate these potential advantages and disadvantages and to what extent they impact overall satisfaction with PD therapy and clinical outcomes. Therefore, a better understanding of what patients like and dislike about their PD therapy may help inform those faced with a dialysis modality decision and help prioritize strategies to improve the PD patient experience, thereby potentially increasing PD uptake and extending technique survival.

Based on responses to a standardized patient questionnaire (PQ), we analyzed data from the Peritoneal Dialysis Outcomes and Practice Patterns Study (PDOPPS) to investigate: 1) patients’ perspectives of PD, including what they consider to be advantages and disadvantages of therapy; and 2) how patient outcomes differed based on their views regarding PD therapy.

## Methods

### Data source and variables

The PDOPPS is an international prospective cohort study in collaboration with the International Society of Peritoneal Dialysis (ISPD) [17]. Patients ≥18 years of age receiving chronic PD are selected randomly from national samples of PD facilities. This analysis includes data from Australia/New Zealand (ANZ), Canada, Japan, Thailand, the United Kingdom (UK), and the US from 2014 to 2017. Study details are provided at https://www.dopps.org/OurStudies/PeritonealDialysisPDOPPS.aspx [17]. Data are collected using uniform and standardized data collection tools, procedures, and processes implemented across the Dialysis Outcomes and Practice Patterns Study (DOPPS) Program. The PDOPPS was approved by a central institutional review board (IRB) in the US, with IRB study approval and patient consent obtained for each patient, as required by national and local ethics committee regulations. Data from US patients receiving care at large dialysis organization (LDO) sites are imported from electronic health records; data from non-LDO US and non-US patients were obtained from manual medical chart abstraction and entered into a web-based data collection tool.

Patient-reported advantages and disadvantages of PD were collected using the PDOPPS PQ, which was mailed to each facility participating in the PDOPPS. All patients who were consented into the PDOPPS were then asked by the facility’s research coordinator or nurse to complete the questionnaire at the time the patient visited the facility for their routine visit. Completing this was voluntary, and patients were able to participate in the study without completing the questionnaire. Participants were included in this analysis if they rated at least 10 of the 17 queried aspects of their PD experience in the PQ. Of the 5274 patients who received a PQ, 2899 (55%) returned the questionnaire, of whom 139 (3%) rated fewer than 10 of the 17 queried aspects of their PD experience. As a result, 2760 (52%) PD patients were included in this analysis. Forty-four patients filled out the PQ after follow-up ended, and therefore, were excluded in the death/transition to HD analysis. Patients excluded from analysis were slightly younger (mean age 58.6 vs. 60.9 years), had shorter PD vintage (mean 1.34 vs. 1.99 years), and were more likely to be on automated PD (APD; 74% vs. 62%) (Additional file 1: Table S1).

**Fig. 1:**
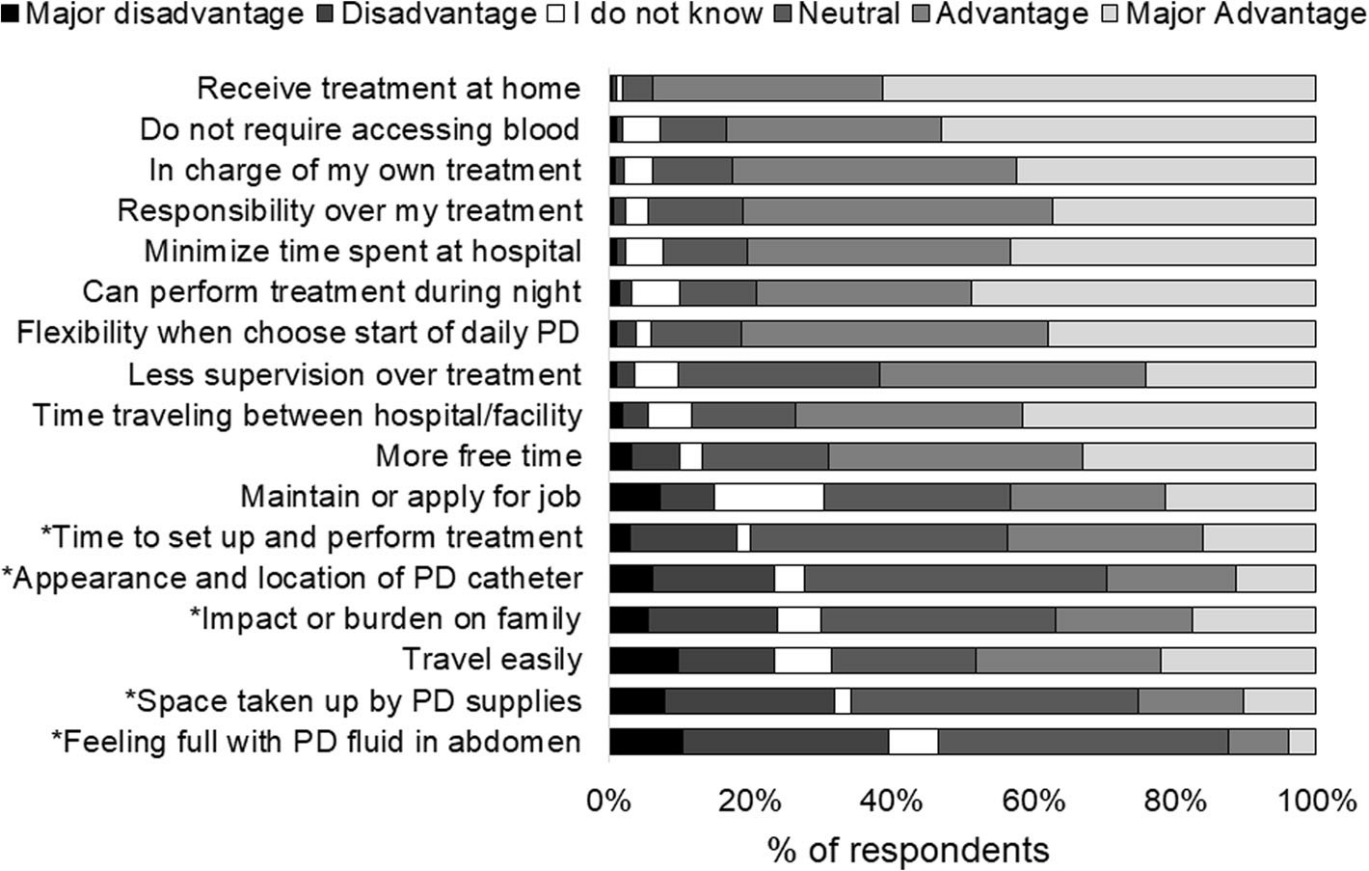
Distribution of responses to 17 questions regarding “To what extent do you feel the following aspects of your peritoneal dialysis treatments are an advantage or disadvantage?” *Indicates the aspects the authors had expected to be disadvantages of peritoneal treatment

Patients were asked in the PQ, “To what extent do you feel the following aspects of your PD treatments are advantages or disadvantages?” (see Fig. 1 for a list of all 17 items). Possible responses to each item were measured on a 5-category ordinal scale: major advantage (coded as + 2), advantage (coded as + 1), neither an advantage nor a disadvantage (0), disadvantage (− 1), or major disadvantage (− 2); or patients could answer “I do not know.” For purposes of this analysis, we combined “I do not know” and “neither an advantage nor a disadvantage” and labeled this as “neutral” (coded as 0). To quantify each patient’s overall perspective regarding PD, an advantage/disadvantage score (ADS) was computed by averaging their response scores and categorizing into four groups: -2 ≤ ADS < 0 (overall disadvantageous); 0 ≤ ADS < 0.5 (slightly advantageous); 0.5 ≤ ADS < 1 (moderately advantageous); and 1 ≤ ADS ≤2 (most advantageous).

In the same questionnaire, we also collected data on other patient-reported outcomes (PROs), including: (i) physical component summary (PCS) and mental component summary (MCS) scores derived from the short form (SF)-12, a subset of the Kidney Disease Quality of Life (KDQOL)-36 questionnaire [18], where lower scores indicated worse QOL; and (ii) depressive symptoms, assessed by the 10-item version of the Center for Epidemiological Studies Depression Screening Index (CES-D) [19]. From these three variables, we defined three binary outcomes: (i) PCS < 32; (ii) MCS < 40; and (iii) CES-D ≥ 10. The cutoff points for PCS and MCS were chosen based on the lowest quartile of the distribution of scores in the study population, and CES-D ≥ 10 was chosen as a positive screen for depressive symptoms based on prior literature [19].

### Statistical analysis

We reported patient characteristics of the total study population by ADS group. We estimated associations between the ADS (exposure) and PROs using logistic regression with generalized estimating equations, assuming an exchangeable working correlation to account for clustering within facilities [20]. Associations were measured as odds ratios (OR) with 95% confidence intervals (CI), adjusted for the following potential confounders: country, an indicator (yes/no) of receipt of dialysis within a US LDO, age, sex, body mass index (BMI), time on PD, 13 summary comorbid conditions (Table 1), serum albumin, 24-h urine volume, and previous HD treatment.

**Table 1:** Patient characteristics by ADS

	Overall	ADS			
Patient characteristic	–	-2 ≤ ADS < 0	0 ≤ ADS < 0.5	0.5 ≤ ADS < 1	1 ≤ ADS ≤2
N patients	2760	181	667	959	953
Age, years	60.9(14.2)	59.0(13.4)	59.7(14.3)	61.2(14.4)	61.8(14.1)
Sex, % male	59%	61%	64%	57%	56%
BMI, kg/m^2^	26.8(6.1)	25.7(5.5)	26.3(6.0)	27.1(6.2)	27.2(6.0)
PD vintage, years	1.99(2.24)	1.80(2.02)	2.04(2.59)	1.94(2.10)	2.04(2.15)
PD modality, % APD	62%	43%	56%	64%	67%
Day dwell, %	66%	71%	70%	67%	60%
Comorbid conditions (%)					
Coronary artery disease	21%	17%	21%	22%	21%
Cancer (non-skin)	11%	8%	11%	10%	13%
Other cardiovascular disease	14%	10%	13%	15%	14%
Cerebrovascular disease	10%	11%	11%	10%	9%
Congestive heart failure	15%	18%	14%	15%	14%
Diabetes	44%	51%	45%	42%	43%
Gastrointestinal bleeding	2%	2%	2%	2%	2%
Hypertension	91%	93%	92%	89%	90%
Lung disease	5%	5%	5%	6%	5%
Neurologic disease	4%	8%	4%	4%	4%
Psychiatric disorder	12%	13%	12%	13%	12%
Peripheral vascular disease	13%	9%	15%	11%	13%
Gangrene/recurrent cellulitis	2%	3%	2%	1%	1%
Peritonitis in last 4 months, %	8%	10%	8%	9%	7%
Albumin, g/dL	3.45(0.56)	3.28(0.60)	3.42(0.57)	3.47(0.53)	3.47(0.56)
24-h urine volume, L	0.95(0.76)	0.74(0.59)	0.92(0.77)	0.96(0.76)	1.00(0.78)
Prescribed therapy volume, L/day	7.92(3.92)	7.72(3.24)	7.82(3.70)	7.94(4.09)	8.02(4.01)
Peritoneal Kt/V urea	1.39(0.53)	1.37(0.48)	1.42(0.52)	1.37(0.50)	1.40(0.56)
Treated with HD treatments^a^, %	42%	39%	44%	39%	45%
Require help setting up/performing PD treatments					
Never	69%	56%	64%	70%	75%
Some of the time	17%	22%	21%	16%	14%
All of the time	14%	22%	15%	13%	11%
Living arrangement					
Lives alone	14%	17%	14%	15%	14%
Lives with spouse, family, or friends	83%	77%	84%	83%	84%
Nursing home, institution, or assisted living unit	1%	3%	1%	0%	1%
Unknown	2%	2%	1%	2%	1%
Homeless	0%	1%	0%	0%	0%

^a^Facility-based or hospital-based HD treatments. Mean (standard deviation) or % shown

Abbreviations: *ADS* advantage/disadvantage score, *APD* automated peritoneal dialysis, *BMI* body mass index, *HD* hemodialysis, *PD* peritoneal dialysis

Cox regression was used to estimate the associations between ADS and three time-to-event outcomes: all-cause mortality; permanent transition to HD; and a composite outcome of mortality or permanent transition to HD, whichever came first. Permanent transfer to HD was defined as deemed permanent transfers or temporary transfers from PD to HD that did not return to PD within 12 weeks (84 days). Follow-up time began upon completion of the PQ and was left-truncated from the start of PDOPPS study enrollment for analyses. Follow-up ended at the event of interest (i.e., death and/or technique failure) or, if no event occurred, 7 days after leaving the facility due to transfer or change in renal replacement therapy modality, transplantation, end of the study phase, or the most recent date of data availability (January 2018). If a patient died within 7 days of permanent transfer to HD, this patient would be counted as both a permanent transfer to HD and a death. Models were stratified by country and US LDO, accounted for facility clustering using robust sandwich covariance estimators; estimated hazard ratios (HR) and 95% CIs were adjusted for the same covariates as listed above.

To better understand which components of the ADS had the biggest impact on outcomes, for items rated as a disadvantage by at least 10% of patients, we dichotomized the responses (major disadvantage and disadvantage vs. major advantage, advantage, and neutral) and used the same modeling approach to examine their individual associations with mortality, transition to HD, PCS score, MCS score, and CES-D.

The *P* values were corrected for multiple hypothesis testing using the Benjamini-Hochberg procedure [21]. For primary analyses, missing covariate values were imputed multiply using the Sequential Regression Multiple Imputation Method by IVEware [22]. Results from 20 imputed data sets were combined for the final analysis using Rubin’s formula [23]. The proportions of missing data were < 10% for all imputed covariates, except for BMI (13%) and 24-h urine volume (43%). All analyses used SAS software, version 9.4 (SAS Institute, Inc).

## Results

### Patient-reported advantages and disadvantages of PD

Figure 1 summarizes how patients rated each of the 17 items related to their perceived advantages and disadvantages of PD. The factor most commonly perceived as an advantage (i.e., advantage or major advantage) was “receive treatment at home” (94% of respondents), followed by “do not require accessing of blood” (84%). The most commonly rated disadvantage (i.e., disadvantage or major disadvantage) of PD treatment was “feeling a full or bloated sensation with my PD fluid in my abdomen” (39%), followed by “space taken up by PD supplies” (32%).

**Fig. 2:**
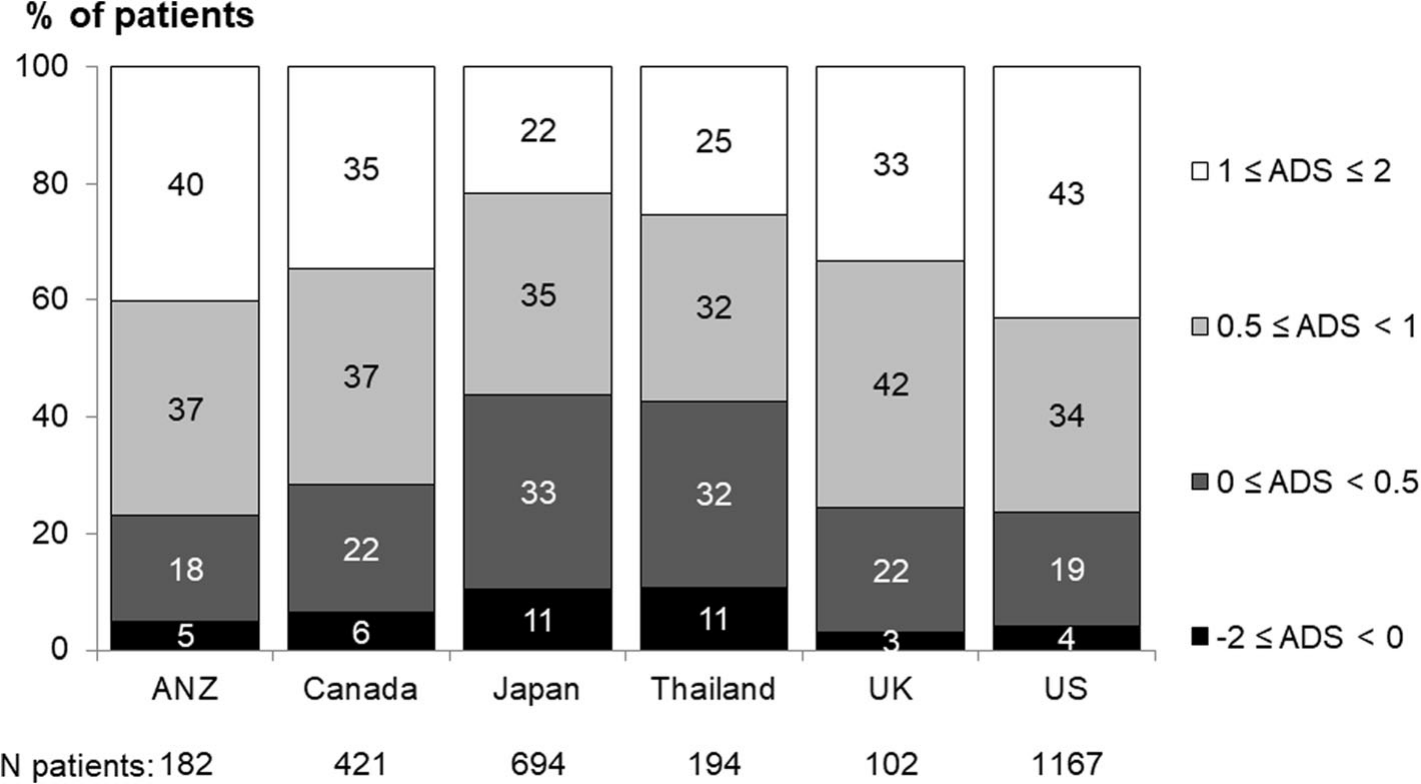
Distribution of ADS, by country. Patient responses are coded −2 (major disadvantage) to + 2 (major advantage). A higher score, calculated from this Likert scale, reflects a more favorable perception of PD. “I do not know” was treated the same as “neither advantage nor disadvantage”

Figure 2 shows the distribution of the ADS by country. Japan and Thailand had the highest proportion of patients with a negative rating (ADS < 0, 11% within both countries), compared with 3–6% elsewhere. The majority of patients had a positive rating; 22–43% with ADS ≥1; and 32–42% with 0.5 ≤ ADS < 1.

### Patient characteristics by ADS

Table 1 shows patient demographic and clinical factors by ADS category. The median ADS was 0.76 (interquartile range [IQR]: 0.41, 1.12), and only 7% had an ADS < 0 (overall negative perception of PD). Patients with a lower ADS were younger (mean age 59.0 years for ADS < 0 vs. 61.8 years for 1 ≤ ADS ≤2), had higher prevalence of diabetes (51% of patients with ADS < 0 vs. 42–45% in other ADS groups), lower albumin (3.28 g/dL for ADS < 0 versus 3.42–3.47 in other ADS groups), and lower residual kidney function (24-h urine volume 0.74 L vs. 0.92–1.00 L). Patients with the lowest ADS scores needed more assistance to help set up and perform PD treatments.

### ADS and adverse clinical outcomes

**Fig. 3:**
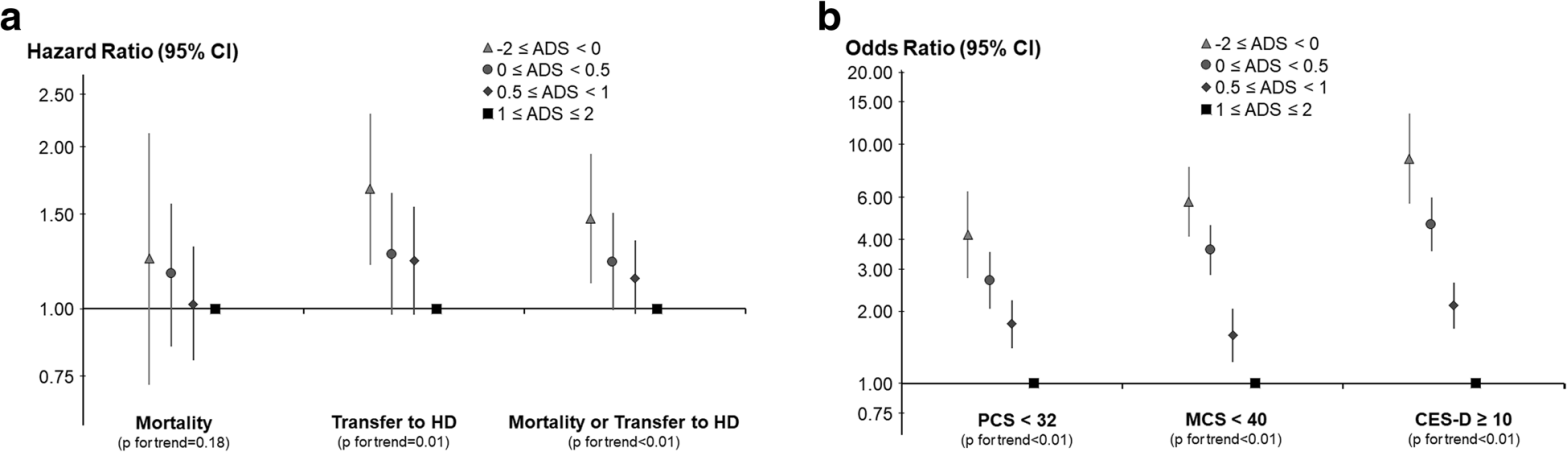
Associations (adjusted HRs and 95% CIs) between the ADS and: **a** all-cause mortality, transition to HD, and the composite outcome (mortality or transition to HD); and (**b**) measures of poor QOL and depression symptoms. The ADS was computed for each patient, where patient responses are coded − 2 (major disadvantage) to + 2 (major advantage). A higher ADS reflects a more favorable perception of PD. PCS and MCS scores were derived from the SF-12, with lower scores indicating worse QOL, and depressive symptoms were assessed by the 10-item version of the CES-D, where CES-D ≥ 10 was a positive screen for depressive symptoms. All models adjusted for the following potential confounders: age, sex, BMI, time on PD, 13 summary comorbid conditions (Table 1), serum albumin, 24-h urine volume, and previous HD treatment. Models for: **a** were stratified by country and US LDO; and for (**b**) were additionally adjusted for country and US LDO

The median length of follow-up was 15.2 months (IQR: 8.0–24.5 months). During follow-up, 339 (12%) patients died, 553 (20%) permanently switched to HD, and 886 composite events (death or transition to HD) were recorded. Compared with an ADS ≥1, patients with an overall negative perception of PD (ADS < 0) were most likely to transition to HD (HR = 1.67; 95% CI: 1.21, 2.30) (Fig. 3a). There was little association between ADS and all-cause mortality.

### ADS and additional PROs

Nearly 21% of patients with an ADS < 0 reported that they regretted the decision to start dialysis, compared with only about 5% of patients with an ADS ≥1. Patients with an ADS < 0 were also more likely to have restless sleep, as 28% had restless sleep most of the time (5–7 days), compared with 13% of patients with an ADS ≥1. Particularly, patients with an ADS < 0 on APD were more often bothered by restless sleep (34% had restless sleep most of the time) compared with patients with an ADS < 0 on continuous ambulatory PD (CAPD; 22%). A slightly higher percentage of patients with an ADS < 0 had peritonitis in the prior 4 months when compared with patients with an ADS ≥1 (10% vs. 7%). Lower ADS was associated with lower PCS and MCS scores of the KDQOL and higher CES-D scores. Compared with patients with ADS ≥1, the adjusted ORs (95% CI) for patients with ADS < 0 were 8.68 (5.64, 13.4) for CES-D score ≥ 10, 5.74 (4.09, 8.04) for MCS < 40, and 4.18 (2.75, 6.34) for PCS < 32 (Fig. 3b).

### Association between top eight disadvantage items and outcomes

**Fig. 4:**
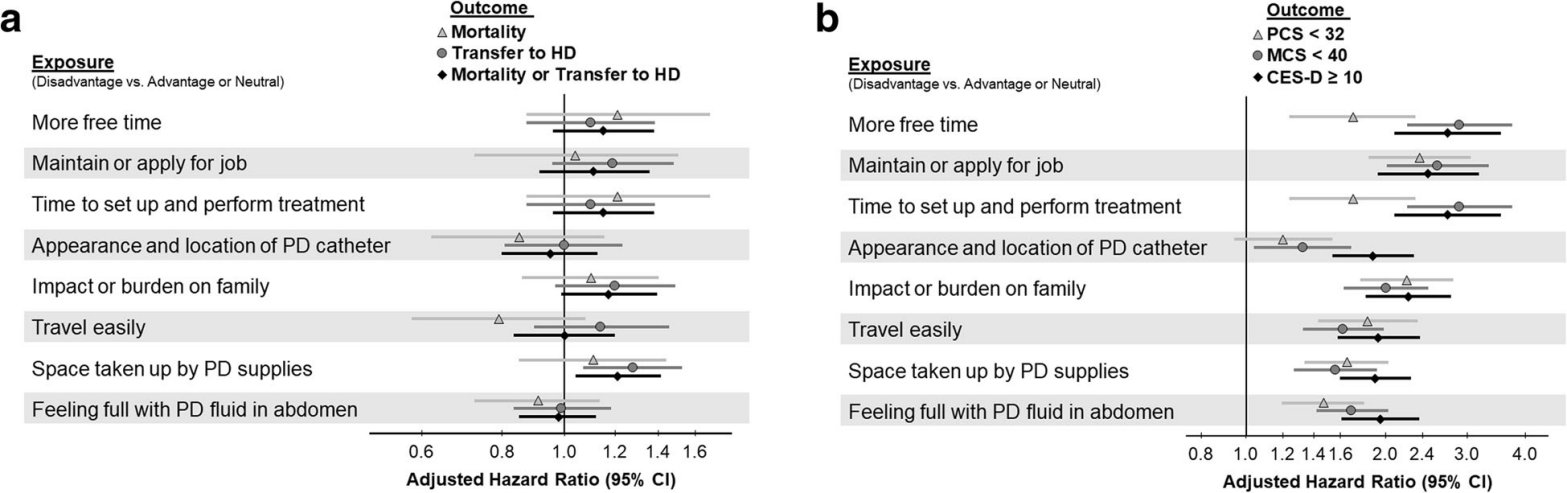
Association between the reporting of an item as a disadvantage and: **a** subsequent hazard of death, HD transition, or both; and (**b**) measures of poor QOL and depression for each of the eight items, in which more than 10% of patients scored that item as a disadvantage of PD. The reference group consisted of patients who reported the item as an advantage or neutral. PCS and MCS scores were derived from the SF-12, with lower scores indicating worse QOL, and depressive symptoms were assessed by the 10-item version of the CES-D, where CES-D ≥ 10 was a positive screen for depressive symptoms. All models adjusted for the following potential confounders: age, sex, BMI, time on PD, 13 summary comorbid conditions (Table 1), serum albumin, 24-h urine volume, and previous HD treatment. Models for: **a** were stratified by country and US LDO; and for (**b**) were additionally adjusted for country and US LDO. Abbreviations: *ADS* advantage/disadvantage score, *BMI* body mass index, *CES-D* Center for Epidemiological Studies Depression Screening Index, *CI* confidence interval, *HD* hemodialysis, *HR* hazard ratio, *LDO* large dialysis organization, *MCS* mental component summary, *PCS* physical component summary, *PD* peritoneal dialysis, *QOL* quality of life, *SF* short form, *US* United States

There were eight individual items rated as a disadvantage by more than 10% of patients. Each item was inconsistently and only weakly associated with mortality, transition to HD, or the composite outcome (Fig. 4a), and this is consistent when *p* values were corrected using the Benjamini-Hochberg procedure. The item most strongly associated with transition to HD was “space taken up by PD supplies” (HR = 1.28; 95% CI: 1.07, 1.53), followed by “impact or burden on family” (HR = 1.20; 95% CI: 0.97, 1.49), and “maintain or apply for job” (HR = 1.19; 95% CI: 0.96, 1.48). Select patient charateristics associated with perception of “space taken up by PD supplies” are shown in Additional file 1: Table S2. The reporting of each of the eight items as a disadvantage was positively associated with poor QOL scores and a high depression score (Fig. 4b).

## Discussion

In this large, international study evaluating patient perceptions of PD, there were four principal findings. First, PDOPPS patients in all countries reported many more advantages of PD than disadvantages, with a median ADS of 0.76. Second, we found that patients with an overall negative perception of PD were more likely than patients with a positive perception to transfer to HD. Third, among individual components of the ADS, we found that the two most frequently reported disadvantages were “feeling full with PD fluid in abdomen” and “space taken up by PD supplies”; however, only the latter perceived disadvantage was also associated with transfer to HD. Fourth, patients with more negative perceptions of PD were more likely to have worse QOL scores and more depressive symptoms.

In a study validating the Customer Satisfaction Questionnaire developed by Fresenius Medical Care, the composite satisfaction score was found to be a good overall measure of patient satisfaction in PD care, but associations of subscale domains with the total score suggested that efforts focused on improving specific aspects might be more effective in increasing patient satisfaction [24]. Hence, we evaluated individual components of the ADS and found that “impact or burden on family,” “maintain or apply for job,” and “space taken up by PD supplies” were associated with transfer to HD. While the opportunity for employment continues to be a perceived advantage of PD (compared with HD), patients may view “maintain or apply for a job” as a disadvantage if they are comparing this with employment prior to starting dialysis (vs. what employment while on HD was or would be like), or perhaps if the ability to “maintain or apply for a job” did not live up to their expectations of the freedom they were counseled to expect with PD. While the loss of employment has been shown to be similar after initiation of HD or PD [25], studies have also shown a significantly higher loss of employment with HD, compared with PD [26]. However, any type of dialysis is likely to be somewhat restrictive, and the benefit of maintaining or applying for a job while on PD may be more of a theoretical advantage. “Space taken up by PD supplies” was the second most commonly rated disadvantage, underscoring the impact of the space required to store PD supplies. Lack of space at home is a frequently-reported barrier among prevalent HD patients when even simply considering home dialysis [27]. While we did not suspect an association of “space taken up by PD supplies” with mortality, we did hypothesize the association with transfer to HD, given that the outcome of transfer to HD is generally more likely to be patient-driven, compared with the outcome of mortality.

It is reasonable to assume that patients are more likely to view this storage space aspect negatively if their living space is small, especially if they have to share their living space. The average residential floor space per capita in the UK is 356 square feet (33.1 m^2^) versus 832 square feet (77.3 m^2^) in the US [28], and likely accounts, in part, for the higher percentage of patients in the UK who viewed “space taken up by PD supplies” as a disadvantage than in the US (Additional file 2: Figure S1). Patients with larger prescribed fluid volumes were more likely to view “space taken up by PD supplies” as a disadvantage (Additional file 1: Table S2), and strategies that minimize total PD fluid needs may be advantageous. However, it is essential that patient care not be compromised by reducing treatment volumes or omitting day-time dwells in an attempt to decrease total PD fluid needs, as this may lead to inadequate dialysis with impaired sodium (and thereby fluid) removal and inadequate middle molecule clearance. While lower dialysate dwell volumes could potentially be advantageous in the new patient, using incrementally larger volumes as needed with the decline of residual kidney function over time, another strategy could be more frequent delivery of supplies, which would reduce the number of boxes in the home at any one time, thereby minimizing the space required for storage without reducing the fluid prescription. Additionally, there is likely to be an emotional component related to viewing “space taken up by PD supplies” as a disadvantage, since the large boxes that occupy space in the home possibly serve as a reminder to the patient that they are living with a chronic disease. Novel technologies may soon enable patients to create dialysate fluid on-site, thereby reducing storage requirements. Future studies will be needed to evaluate whether and to what extent reducing the burden of solution storage may minimize negative perceptions of PD and, therefore, possibly prolong PD technique survival.

Patients with a less favorable perception of PD were younger, had a lower BMI, and had lower 24-h urine volumes (Table 1). Patients with lower BMIs may be more sensitive to their dwell volumes, possibly feeling full more easily, and, therefore, may view PD more negatively. Additionally, those with lower 24-h urine volumes may try to achieve greater peritoneal ultrafiltration with greater fill volumes, thereby further contributing to a feeling of fullness. We found that patients using APD tended to view PD more positively than those using CAPD, similar to previous findings [29]. Patients using APD accomplish the majority of their therapy at night while sleeping, allowing more flexibility with their time during the day. Similarly, we found those with a “day dwell” viewed PD more negatively, as the effect of gravity while patients are upright likely causes more discomfort. The lowest ADS category had a higher percentage of patients who required help setting up and performing PD treatments; this supports previous reports, whereby PD patients commonly listed “troubling other people” as a main disadvantage of PD [15].

In the analysis of PROs, lower ADS scores – suggesting a more negative perception of PD – were associated with lower scores of physical and mental health and increased symptoms of depression. It is possible that the direction of this association may be such that the negative perception of PD led to inferior QOL and greater depression. Equally plausible is that this association may be the result of patients with impaired QOL and depression at baseline, which impacted their perception of PD. Previous studies have demonstrated an independent association between depression and increased mortality risk in dialysis patients [30], as well as an independent association between depression and peritonitis rates [31], which is a common cause of transfer to HD [32].

This study should be viewed in the context of the following limitations. First, given the observational study design, we cannot rule out residual confounding due to unmeasured risk factors or model misspecification. For example, we did not collect information on the sizes and locations of patients’ homes, which may have explained some of our findings. Second, as previously mentioned, since the analyses between ADS and the PROs of MCS, PCS, and CES-D scores were cross-sectional, the possibility of reverse causation limits the ability to assume a causal relationship. Third, the cross-sectional analyses are also susceptible to selection bias because the survey outcomes could have influenced the selection of subjects, which is especially true in this study, given that the survey response rate was only slightly better than 50%. However, as shown in Additional file 1: Table S1, there were no large differences between survey respondents and non-respondents. Fourth, the high proportion of PD patients reporting favorable views of PD might have been exaggerated due to the possible tendency of patients to answer those survey questions in ways they thought were expected or desired by the investigators. Finally, while most validated questionnaires currently assess QOL among PD patients, one validated survey assigns priority to aspects of the dialysis patient experience based on patient responses, but only a single aspect relates to PD specifically: “immediate help in case of peritonitis” [33]. Although the survey questions of our 17-item questionnaire were generated based on expert consensus, many of the items overlap with what long-term PD patients have identified as reasons for choosing PD [34]. However, while little prior literature or psychometrics informed the content of our survey, this will serve as a stepping stone for future work in developing a validated questionnaire, using more patient engagement, to meaningfully assess the balance between patient-perceived PD advantages and disadvantages.

## Conclusions

This is the largest study to-date to include PROs on PD patients, and we found associations between an overall negative perception of PD therapy and a higher rate of transferring to HD, worse QOL scores, and more depressive symptoms. Moreover, patients who viewed “space taken up by PD supplies” as a disadvantage had a higher rate of transferring to HD. Our findings will assist nephrologists and members of the end-stage kidney disease (ESKD) care team to better provide more informed counseling with quantifiable advantages and disadvantages to patients considering PD as their dialysis modality. Additionally, given the possible influence of reverse causation in the cross-sectional findings of this paper, not only could improving the perceived disadvantages of therapy improve QOL and depression, but efforts directed at enhancing support for patients’ physical and mental well-being and improving depression symptoms may help improve patients’ perceptions of PD therapy. Future studies are needed to determine the extent to which the expected advantages and disadvantages of PD in pre-ESKD patients are similar to the perceptions of those patients after starting PD, as this will allow for more accurate and personalized counseling of ESKD patients considering PD. Finally, given that “space taken up by PD supplies” is a commonly perceived disadvantage, and that this perception is associated with an increased likelihood of transferring to HD, modifying therapy with new technologies, such as on-site PD fluid creation, may positively affect the patient’s experience and is worth further investigation.

## Additional files

Additional file 1: **Table S1.** Comparison of patient characteristics for those included versus excluded from the study population. **Table S2.** Patient characteristics by answer responses for “space taken up by PD supplies”. (DOCX 22 kb)

Additional file 2: Distribution of responses to “space taken up by PD supplies,” by country. (TIF 78 kb)

## Abbreviations

ADS: Advantage/disadvantage score
ANZ: Australia/New Zealand
APD: Automated peritoneal dialysis
BMI: Body mass index
CAPD: Continuous ambulatory PD
CES-D: Center for Epidemiological Studies Depression Screening Index
CI: Confidence interval
DOPPS: Dialysis Outcomes and Practice Patterns Study
HD: Hemodialysis
HR: Hazard ratio
IQR: Interquartile range
IRB: Institutional review board
ISPD: International Society of Peritoneal Dialysis
KDQOL: Kidney Disease Quality of Life
LDO: Large dialysis organization
MCS: Mental component summary
PCS: Physical component summary
PD: Peritoneal dialysis
PDOPPS: Peritoneal Dialysis Outcomes and Practice Patterns Study
PQ: Patient questionnaire
PRO: Patient-reported outcome
QOL: Quality of life
SF: Short form
UK: United Kingdom
US: United States
