# Supplementary material for: Patient-reported advantages and disadvantages of peritoneal dialysis: results from the PDOPPS
Source: BMC Nephrol. 2019 Apr 2;20:116. doi: 10.1186/s12882-019-1304-3 (PMC6446371; doi:10.1186/s12882-019-1304-3)
Supplement: Supplementary file 1 — Table S1. Comparison of patient characteristics for those included versus excluded from the study population. Table S2. Patient characteristics by answer responses for “space taken up by PD supplies”. (DOCX 22 kb) [file 12882_2019_1304_MOESM1_ESM.docx]

**Additional file 1**

**Patient-Reported Advantages and Disadvantages of Peritoneal Dialysis: Results from the PDOPPS**

Nidhi Sukul, Junhui Zhao, Douglas S. Fuller, Angelo Karaboyas, Brian Bieber, James A. Sloand, Lalita Subramanian, David W. Johnson, Matthew J. Oliver, Kriang Tungsanga, Tadashi Tomo, Rachael L. Morton, Hal Morgenstern, Bruce M. Robinson, Jeffrey Perl, on behalf of the clinical application of PD therapy working group

**SUPPLEMENTAL TABLES**

**Supplemental Table S1. Comparison of patient characteristics for those included versus excluded from the study population.**

| **Patient characteristic** | **Included** | **Excluded** |
| --- | --- | --- |
| N patients | 2760 | 2514 |
| Age, years | 60.9(14.2) | 58.6(15.5) |
| Sex, % male | 59% | 61% |
| BMI, kg/m^2^ | 26.8(6.1) | 28.1(6.4) |
| PD vintage, years | 1.99(2.24) | 1.34(1.63) |
| PD modality, % APD | 62% | 74% |
| Day dwell, % | 66% | 52% |
| Comorbid conditions (%) |  |  |
| Coronary artery disease | 21% | 22% |
| Cancer (non-skin) | 11% | 9% |
| Other cardiovascular disease | 14% | 13% |
| Cerebrovascular disease | 10% | 6% |
| Congestive heart failure | 15% | 12% |
| Diabetes | 44% | 50% |
| Gastrointestinal bleeding | 2% | 2% |
| Hypertension | 91% | 87% |
| Lung disease | 5% | 6% |
| Neurologic disease | 4% | 4% |
| Psychiatric disorder | 12% | 14% |
| Peripheral vascular disease | 13% | 14% |
| Gangrene/recurrent cellulitis | 2% | 2% |
| Albumin, g/dL | 3.45(0.56) | 3.39(0.55) |
| 24-hour urine volume, L | 0.95(0.76) | 0.95(0.77) |
| Prescribed therapy volume, L/day | 7.92(3.92) | 7.98(4.41) |
| Peritoneal Kt/V urea | 1.39(0.53) | 1.47(0.53) |

Mean (standard deviation) or % shown.

Abbreviations: APD, automated peritoneal dialysis; BMI, body mass index; PD, peritoneal dialysis.

**Supplemental Table S2. Patient characteristics by answer responses for “space taken up by PD supplies”.**

|  | **Space taken up by PD supplies** | | |
| --- | --- | --- | --- |
| **Patient characteristic** | **Disadvantage** | **Neutral** | **Advantage** |
| N patients | 866 | 1171 | 684 |
| Age, years | 57.3(14.8) | 61.7(14.0) | 63.8(13.1) |
| Sex, % male | 57% | 59% | 61% |
| BMI, kg/m2 | 27.5(6.3) | 26.8(6.0) | 26.2(5.8) |
| Diabetes, % | 44% | 44% | 50% |
| PD vintage, years | 1.76(2.06) | 1.99(2.18) | 2.19(2.34) |
| PD modality, % APD | 72% | 60% | 53% |
| Day dwell, % | 60% | 67% | 69% |
| Albumin, g/dL | 3.46(0.56) | 3.46(0.55) | 3.41(0.56) |
| 24-hour urine volume, L | 0.95(0.76) | 0.98(0.78) | 0.90(0.73) |
| Prescribed therapy volume, L/day | 8.39(4.23) | 7.84(3.82) | 7.60(3.68) |
| Peritoneal Kt/V urea | 1.42(0.57) | 1.35(0.49) | 1.43(0.53) |
| Living arrangement |  |  |  |
| Lives alone | 17% | 12% | 15% |
| Lives with spouse, family, or friends | 81% | 85% | 82% |
| Nursing home/institution or assisted living unit | 0% | 1% | 1% |
| Unknown | 2% | 1% | 2% |

Mean (standard deviation) or % shown.

Abbreviations: APD, automated peritoneal dialysis; BMI, body mass index; PD, peritoneal dialysis.
