# Supplementary figures and images for: Patient-reported advantages and disadvantages of peritoneal dialysis: results from the PDOPPS
Source: BMC Nephrol. 2019 Apr 2;20:116. doi: 10.1186/s12882-019-1304-3 (PMC6446371; doi:10.1186/s12882-019-1304-3)

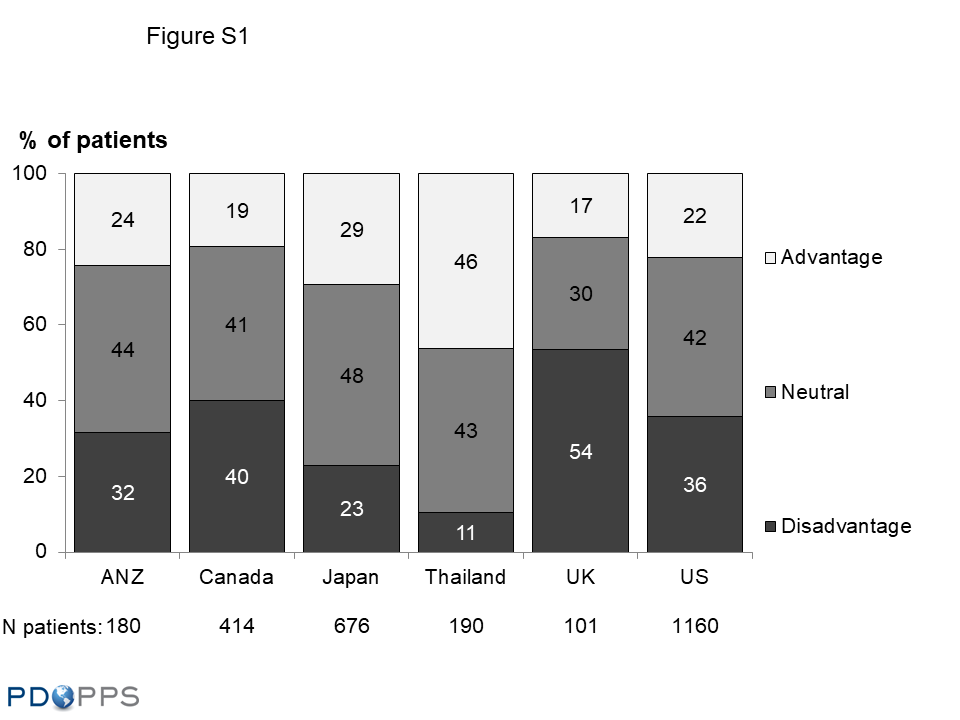

Supplement: Supplementary file 2 — Distribution of responses to “space taken up by PD supplies,” by country. (TIF 78 kb) [file 12882_2019_1304_MOESM2_ESM.tif]
